# Impact of coronary computed tomography angiography-derived fractional flow reserve based on deep learning on clinical management

**DOI:** 10.3389/fcvm.2023.1036682

**Published:** 2023-02-02

**Authors:** Yueying Pan, Tingting Zhu, Yujijn Wang, Yan Deng, Hanxiong Guan

**Affiliations:** ^1^Department of Radiology, Tongji Medical College, Tongji Hospital, Huazhong University of Science and Technology, Wuhan, China; ^2^Depatment of Pulmonary and Critical Care Medicine, Tongji Medical College, Tongji Hospital, Huazhong University of Science and Technology, Wuhan, China

**Keywords:** coronary artery disease, coronary computed tomography angiography-derived fractional flow reserve, radiation dose, deep learning, DL-FFRCT

## Abstract

**Background:**

To examine the value of coronary computed tomography angiography (CCTA)-derived fractional flow reserve based on deep learning (DL-FFRCT) on clinical practice and analyze the limitations of the application of DL-FFRCT.

**Methods:**

This is an observational, retrospective, single-center study. Patients with suspected coronary artery disease (CAD) were enrolled. The patients underwent invasive coronary angiography (ICA) examination within 1 months after CCTA examination. And quantitative coronary angiography (QCA) was performed to evaluate the area stenosis rate. The CCTA data of these patients were retrospectively analyzed to calculate the FFRCT value.

**Results:**

A total of 485 lesions of coronary arteries in 229 patients were included in the analysis. Of the lesions, 275 (56.7%) were ICA-positive, and 210 (43.3%) were FFRCT-positive. The discordance rate of the risk stratification of FFRCT for ICA-positive lesions was 33.1% (91) and that for ICA-negative lesions was 12.4% (26). 14.6% (7/48) patients with mild to moderate coronary stenosis in ICA have functional ischemia according to FFRCT positive indications. In addition, hemodynamic analysis of severely calcified, occluded, or small (< 2 mm in diameter) coronary arteries by DL-FFRCT is not so reliable.

**Conclusion:**

This study revealed that most patients with ICA negative did not require further invasive FFR. Besides, some patients with mild to moderate coronary stenosis in ICA may also have functional ischemia. However, for severely calcified, occluded, or small coronary arteries, treatment strategy should be selected based on ICA in combination with clinical practice.

## 1. Introduction

Coronary artery disease (CAD) is responsible for 21.1% of all deaths worldwide, and the prevalence is still increasing, making CAD the most common cause of cardiovascular disease mortality (1). Coronary computed tomography angiography (CCTA) is widely used in patients with suspected CAD. In contrast to invasive coronary angiography (ICA), CCTA is non-invasive and does not require hyperemic agent, adenosine, or papaverine (2–4). Unfortunately, CCTA has a high sensitivity and high negative predictive value for CAD detection, but relatively low specificity for diagnosis of CAD (5).

Recently, CCTA-derived fractional flow reserve (FFRCT) has been recommended for evaluating functional severity by utilizing computational fluid dynamics (CFD) to calculate coronary blood pressure (6). Several large-scale, multi-center studies have demonstrated that the results of FFRCT are consistent with those of invasive FFR, and are better than CCTA alone (7–9). A recent meta-analysis of 1,825 patients and 2,731 coronary arteries showed that FFRCT obtained a high diagnostic performance and is a viable alternative to invasive fractional flow reserve (FFR) for detecting coronary ischemic lesions (10). However, a supercomputer is often required for the mostly previous FFRCT analysis, and the calculation process was time-consuming and money-consuming. A recent improvement in the development of FFRCT is the introduction of deep learning algorithm (DL-FFRCT). The DL-FFRCT performs equally in detecting lesion-specific ischemia when compared with the FFRCT approach based on CFD (11).

The purpose of the present study was to explore application of DL-FFRCT on clinical practice and analyze the limitations of DL-FFRCT, so as to clarify the value of DL-FFRCT in the clinical decision-making of CAD patients.

## 2. Materials and methods

### 2.1. Study population

This is an observational, retrospective, single-center study and received institutional review board approval, and the informed consent was obtained from all enrolled patients. Patients with a documented degree of stenosis > 50% on CCTA in a tertiary hospital, Wuhan, Hubei, China, between January 2014 and May 2021, were retrospectively reviewed. The patients underwent ICA examination within 1 month after CCTA examination. Patients with previous coronary artery revascularization, previous myocardial infarction, or previous stroke were excluded. The CCTA data of these patients were retrospectively analyzed to calculate the FFRCT value. The clinical data of patients about gender, age, symptoms, blood pressure, heart rate, left ventricular ejection fraction (LVEF), past medical history, cardiovascular medication history, family history, smoking, alcohol consumption, blood glucose, triglycerides and cholesterol were obtained from medical records.

### 2.2. CCTA acquisition

All patients were examined on a second-generation 320-row CT scanner (Aquilion ONE Vision, Toshiba, Japan), and Discovery CT750 HD (GE Healthcare, Milwaukee, USA) according to guidelines, using prospective ECG-triggered axial scans. All reconstructed images were transferred to a dedicated workstation (ADW 4.7; GE Healthcare, USA). Two radiologists with more than 5 years of experience in cardiovascular CT image diagnosis were used to evaluate CCTA image quality and diagnose the degree of coronary stenosis, and then the sequence with the best image quality was selected for subsequent FFRCT calculation. The degree of stenosis was defined as the ratio of the diameters of the stenotic and reference vessels, and the vessels with a CCTA stenosis over 50% were referred for further evaluation by ICA.

### 2.3. DL-FFRCT

An artificial intelligence deep-learning software prototype (DEEPVESSEL, KEYA Medical, China) was used to calculate FFRCT value in a manner that was blinded to the clinical findings according to the previous researches (12, 13). It utilizes a deep learning algorithm to learn the complex mapping between FFR and the input features derived from the coronary artery anatomical data. The deep learning framework consists of a multi-layer perceptron network (MLP) and a bidirectional multi-layer recursive neural network (BRNN). It not only considers the various features at each vessel location independently by MLP, but also embeds the spatial relationships among coronary artery tree structures through BRNN. Thus, it is able to seamlessly integrate information from all locations in the coronary artery tree to make an accurate calculation. Given a CTA image, the 3D coronary artery model and its centerlines were first extracted and FFRCT values along the centerlines were then calculated using the novel deep learning algorithm mentioned above. The CCTA image data in DICOM format were transferred to the DEEPVESSEL platform and the FFRCT value was obtained. DEEPVESSEL calculated FFRCT value 2 cm distal to coronary stenosis. For long-segment stenosis, FFRCT values were calculated at 2 cm distal to the end of the stenosis. For multiple stenosis, FFRCT values were calculated 2 cm distal to the last stenosis. The threshold value of FFRCT ≤ 0.80 was defined as positive, with excellent accuracy, sensitivity and specificity (14).

### 2.4. ICA procedure

ICA was performed according to societal guidelines and quantitative coronary angiography (QCA) was performed in angiography X-ray equipment (Artis zee III ceiling, SIEMENS Healthineers, Germany) (15). At least two perpendicular projections were selected for the coronary artery lesion site, and the position with the most severe degree of stenosis was selected to quantitatively measure the degree of coronary artery stenosis. The area stenosis rate was evaluated, with stenosis ≥ 75% as a positive ICA indication for revascularization. The treatment strategy also relied on the location of the stenosis, the length of the stenosis, the diameter of the target vessel, and the patients’ treatment intention.

### 2.5. Statistical analysis

The statistical analyses were carried out using IBM SPSS (SPSS Inc., Version 25). The Shapiro-Wilk test was conducted to assess the normality of the quantitative data. Quantitative variables were expressed as mean ± *SD* if normally distributed; while median and inter-quartile range (IQR) was provided for non-normally distributed data. Categorical data are presented as frequency and percentage. And variables were compared using Student’s *t*-test, ANOVA, the Mann-Whitney *U*-test, the Kruskal-Wallis H test, or χ^2^-test as appropriate. The between-methods consistency was examined by kappa test (kappa < 0.4: fair; 0.4–0.6: moderate; 0.6–0.8: substantial; > 0.8: almost perfect). Receiver operating characteristic (ROC) curve of FFRCT was depicted and the area under the receiver operating characteristic curve (AUC) was obtained. Regression analysis was used to find out the factors affecting ICA positivity and the inconsistency between ICA and FFRCT. All statistical tests were two-tailed, and a *P*-value < 0.05 was defined as significant.

## 3. Results

### 3.1. Patient demographics

A total of 604 patients were initially included, including 251 patients with CCTA stenosis < 50%, 39 patients with coronary stents, and 85 patients with motion artifacts of CCTA images that could not be calculated by DL-FFRCT. Finally, a total of 229 patients were included in the present study, all of whom underwent CCTA and ICA examinations, and DL-FFRCT results were derived from CCTA data, of which 181 had positive ICA results. In the patients, a total of 485 coronary arteries had lesions, including 201 left anterior descending (LAD), 135 left circumflex arteries (LCX), and 149 right coronary arteries (RCA). The area stenosis rate was 51 ± 38%. Patients’ demographics and clinical data are summarized in Table 1. Notably, 95 patients presented with angina, non-angina chest pain or dyspnea, 50 patients were admitted with chest tightness and palpitation, which may be atypical symptoms of coronary heart disease. The remaining 84 patients were admitted for other reasons, and routine electrocardiogram examination was found to be abnormal, so the cardiologist recommended CCTA examination.

**TABLE 1:** Demographics of the enrolled patients.

Total	229
Gender	*n* (%)
Male	162 (70.7%)
Female	67 (29.3%)
**Age**	**59.4 ± 10.3 years**
Symptom	*n* (%)
Angina	71 (31.0%)
Non-angina chest pain	19 (8.3%)
Dyspnea	5 (2.2%)
Medical history	*n* (%)
Hypertension	119 (52.0%)
Diabetes mellitus	35 (15.3%)
Hyperlipemia	9 (3.9%)
Cerebral infarction/Hemorrhage	19 (8.3%)
Medication history	*n* (%)
ACEi/ARB	28 (12.2%)
β-blocker	17 (7.4%)
Calcium antagonist	45 (19.7%)
Diuretic	8 (3.5%)
Aspirin	13 (5.7%)
Antilipemic agents	4 (1.7%)
Oral antidiabetic agents/insulin	22 (9.6%)
Myocardial infarction history	4 (1.7%)
Family history of CAD	28 (12.2%)
Smoking history	99 (43.2%)
Drinking history	56 (24.5%)
Blood glucose	6.5 ± 2.6 mmol/L
>6.05	73 (31.9%)
Total cholesterol	4.0 ± 1.1 mmol/L
>5.18	27 (11.8%)
Total triglycerides	2.0 ± 1.4 mmol/L
>1.7	83 (36.2%)
LVEF	*n* (%)
<50%	8 (3.5%)

### 3.2. Risk stratification

**FIGURE 1:**
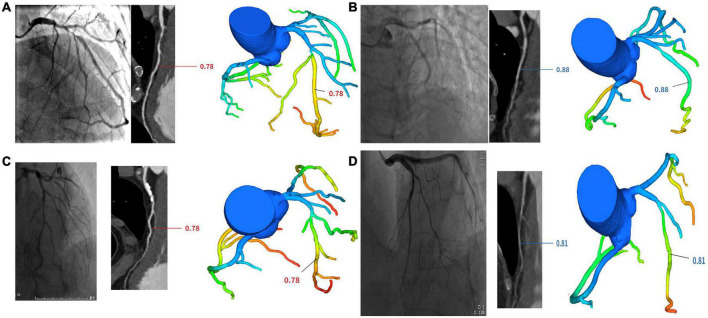
Cases of four groups. **(A)** One case in IpFp group. ICA revealed proximal LAD stenosis 90% and FFRCT showed hemodynamic ischemia due to proximal segment stenosis (FFRCT = 0.78). **(B)** One case in IpFn group. ICA revealed proximal LAD stenosis 85% and FFRCT showed no hemodynamic ischemia (FFRCT = 0.88). **(C)** One case in InFp group. ICA revealed proximal LAD stenosis 70% and FFRCT showed hemodynamic ischemia (FFRCT = 0.78). **(D)** One case in InFn group. ICA revealed proximal LAD stenosis 60% and FFRCT showed no hemodynamic ischemia (FFRCT = 0.81).

The datasets were subdivided into four groups. These groups were defined as (1) positively confirmed by both ICA and FFRCT (IpFp), (2) positively confirmed by ICA but negative by FFRCT (IpFn), (3) negatively confirmed by ICA but positive by FFRCT (InFp), and (4) negatively confirmed by both ICA and FFRCT (InFn) (see Figure 1).

#### 3.2.1. Per-patient analysis

As shown in Table 2, The patient-wise distribution was 146 (63.8%), 35 (15.3%), 7 (3.1%), and 41 (17.9%) patients in the IpFp, IpFn, InFp, and InFn groups, respectively. FFRCT showed a moderate consistence with ICA, with a kappa value of 0.54. Of the patients, 181 (79.0%) were ICA-positive, and 153 (66.8%) were FFRCT-positive. FFRCT results were inconsistent with ICA results in 42 patients. The discordance rate of the risk stratification of FFRCT for ICA-positive patients was 35 (19.3%) and that for ICA-negative patients was 7 (14.6%). Of the 48 ICA negative patients, 41 (85.4%) were also negative for FFRCT, and severe stenosis could be ruled out anatomically and functionally. In other words, most patients with ICA negative did not require further invasive FFR. Given the high consistency between FFRCT and invasive FFR in previous studies, whether the remaining 7 FFRCT-positive patients should receive ICA needs further investigation. Besides, 14.6% (7/48) patients with mild to moderate coronary stenosis in ICA have functional ischemia according to FFRCT positive indications.

**TABLE 2:** Patients and coronaries distribution of four groups.

	IpFp	IpFn	InFp	InFn	Total
Patient	146	35	7	41	229
Kappa					0.54
LAD	107	29	12	53	201
LCX	28	41	5	61	135
RCA	49	21	9	70	149
Coronaries	184	91	26	184	485
Kappa					0.53

Of the 181 ICA-positive patients, 7 patients refused to have stents for economic reasons, 3 patients were given priority in treating other serious diseases (1 patient with bladder cancer, 1 patient with cervical cancer, 1 patient complicated by mediastinal tumor), 1 patient was preceded by mitral valve mechanical valve replacement, 1 patient received medical therapy due to the high risk of poor cardiac function surgery, 5 patients underwent coronary artery bypass surgery, and the rest 164 patients were treated with percutaneous coronary intervention (PCI).

**FIGURE 2:**
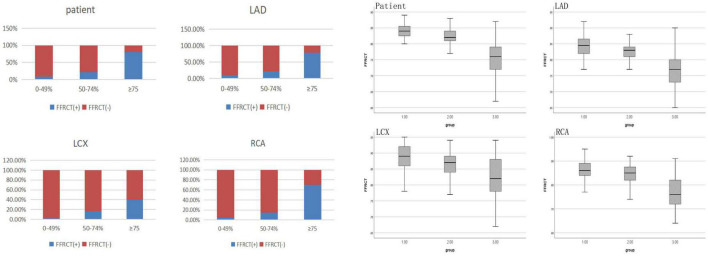
FFRCT values of coronary arteries with different degrees of stenosis in invasive coronary angiography.

As shown in Table 3 and Figure 2, Of the 25 patients with 0–49% stenosis, 2 (8.0%) had positive FFRCT results; in 23 patients with 50–74% stenosis, 5 (21.7%) had positive FFRCT results, and of 181 patients with ≥ 75% stenosis, 146 (80.7%) patients had positive FFRCT results, significantly higher than the proportion of patients with FFRCT positive in the 50–74% stenosis group. The median FFRCT value was 0.84 (0.04) in the 0–49% stenosis group, 0.82 (0.03) in the 50–74% stenosis group, and the median FFRCT value was 0.76 (0.07) in the ≥ 75% stenosis group, significantly lower than that in the 50–74% stenosis group.

**TABLE 3:** FFRCT values of coronary arteries with different degrees of stenosis in coronary angiography.

Patient	*n*	FFRCT (+)	FFRCT (−)	χ	*p*	FFRCT	*z/F*	*p*
0–49%	25	2 (8.0%)	23 (92.0%)	140.23	0.00	0.84 (0.04)	58.62	0.000
50–74%	23	5 (21.7%)*	18 (78.3%)			0.82 (0.03)*		
≥75%	181	146 (80.7%)	35 (19.3%)			0.76 (0.07)		
**LAD**								
0–49%	57	6 (10.5%)	51 (89.5%)	90.93	0.000	0.85 ± 0.033	74.51	0.000
50–74%	36	8 (22.2%)*	28 (77.8%)			0.83 ± 0.028*		
≥75%	136	107 (78.7%)	29 (21.3%)			0.77 ± 0.052		
**LCX**								
0–49%	142	3 (2.1%)	139 (97.9%)	54.40	0.000	0.89 (0.06)	56.42	0.000
50–74%	18	3 (16.7%)*	15 (83.3%)			0.87 (0.06)		
≥75%	69	28 (40.6%)	41 (59.4%)			0.82 (0.10)		
**RCA**								
0–49%	132	6 (4.5%)	126 (95.5%)	150.10	0.000	0.86 ± 0.033	97.95	0.000
50–74%	27	4 (14.8%)*	23 (85.2%)			0.85 ± 0.043*		
≥75%	70	49 (70.0%)	21 (30.0%)			0.77 ± 0.067		

*Significant difference compared with patients with coronary stenosis ≥ 75%.

#### 3.2.2. Per-coronary analysis

As shown in Table 2, analysis of single diseased coronary arteries showed that the distribution of coronary arteries in IpFp, IpFn, InFp, and InFn groups were 184 (37.9%), 91 (18.8%), 26 (5.3%), and 184 (37.9%), respectively. Of the coronary arteries, 275 (56.7%) were ICA-positive, and 210 (43.3%) were FFRCT-positive. The inconsistency rate of the risk stratification of FFRCT for ICA-positive lesions was 33.1% (91) and that for ICA-negative lesions was 12.4% (26), with a total of 117 coronary arteries. FFRCT showed a moderate consistence with ICA, with a kappa value of 0.53.

FFRCT values of coronary arteries with different degrees of stenosis in coronary angiography were shown in Table 3 and Figure 2. DL-FFRCT method has higher sensitivity in detecting severe lesions (≥ 75% by ICA) in LAD in comparison to LCX and RCA (78.7% vs. 40.6%, 78.7% vs. 70.0%).

In detail, of the 136 LAD with ≥ 75% stenosis, 107 (78.7%) patients had positive FFRCT results, significantly higher than the proportion of patients with FFRCT positive in the 50–74% stenosis group 8 (22.2%). The mean FFRCT value of the ≥ 75% stenosis group was significantly lower than that of the 50–74% stenosis group (0.77 ± 0.052 vs. 0.83 ± 0.028, *P* < 0.05). Of the 69 LCX with ≥ 75% stenosis, 28 (40.6%) patients had positive FFRCT results, significantly higher than the proportion of patients with FFRCT positive in the 50–74% stenosis group 3 (16.7%). The median FFRCT value of the ≥ 75% stenosis group was lower than that of the 50–74% stenosis group [0.82 (0.10) vs. 0.87 (0.06), *P* > 0.05] without significance, probably due to the small sample size of 50–74% stenosis group. Of the 70 RCA with ≥ 75% stenosis, 49 (70.0%) patients had positive FFRCT results, significantly higher than the proportion of patients with FFRCT positive in the 50–74% stenosis group 4 (14.8%). The median FFRCT value of the ≥ 75% stenosis group was significantly lower than that of the 50–74% stenosis group (0.77 ± 0.067 vs. 0.85 ± 0.043, *P* < 0.05).

### 3.3. Diagnostic performance of FFRCT for diagnosis of ischemia per-coronary according to ICA

**FIGURE 3:**
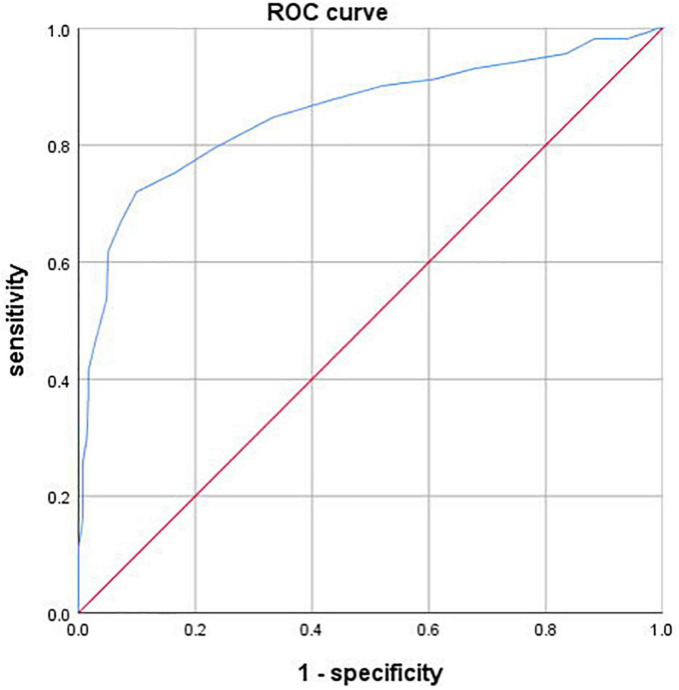
Receiver operating characteristic (ROC) curve of FFRCT. The area under the receiver operating characteristic curve (AUC) was 0.86 (95% CI, 0.82–0.89).

FFRCT demonstrated sensitivity, specificity, positive predictive value and negative predictive value of 0.67 (95% CI, 0.61–0.72), 0.88 (95% CI, 0.82–0.92), 0.88 (95% CI, 0.82–0.92), and 0.67 (95% CI, 0.61–0.72) using ICA as standard. ROC curve of FFRCT was depicted in Figure 3. The on a per-vessel basis FFRCT demonstrated excellent diagnosis performance according to ICA [AUC, 0.86 (95% CI, 0.82–0.89)].

### 3.4. Analysis of the causes of inconsistencies between FFRCT and ICA results

FFRCT, age, gender, LVEF, hypertension, angina, blood glucose, cholesterol, and triglyceride were included in the multivariate Logistics regression analysis. The results showed that only FFRCT had statistically significant effect on ICA positivity (OR = 49.91, 95% CI 12.769–195.07, *P* < 0.001). Furthermore, FFRCT, age, gender, LVEF, hypertension, angina, blood glucose, cholesterol and triglyceride were included to construct a multi-factor Logistics regression equation to analyze the factors affecting the inconsistency between ICA and FFRCT results for all of the patients. The results showed that the above factors had no statistically significant impact on the inconsistency between ICA and FFRCT results. Therefore, we tried to further analyze other possible reasons for the inconsistency between ICA and FFRCT results.

**FIGURE 4:**
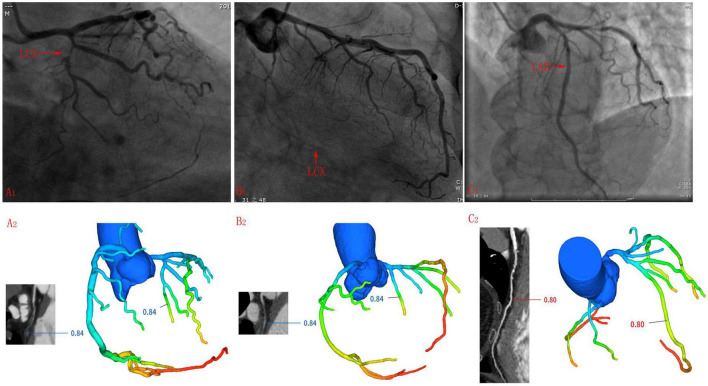
Cases whose ICA results were inconsistent with FFRCT. **(A1,A2)** LCX was too small to reconstruct the distal end during three-dimensional reconstruction before calculating the FFRCT value. **(B1,B2)** The middle segment of the LCX was occluded, so it is impossible to reconstruct the middle and far sections of the LCX when undergoing the three-dimensional reconstruction before calculating the FFRCT value, and the FFRCT value of the near LCX segment was given. **(C1,C2)** Long segment calcification was seen in the mid-LAD segment.

There were 117 (117/485, 24.1%) coronary arteries whose FFRCT results were inconsistent with ICA results, including 43 LADs, 46 LCXs, and 28 RCAs. Among them, the stenosis rate of 13 coronary arteries was 99–100%, that is, subtotal or complete occlusion. When measuring FFRCT value of these patients, the FFRCT value of coronary artery segment before occlusion was measured because the occlusion coronary artery could not be reconstructed (see Figures 4A1, A2). 12 LCXs and 6 RCAs were small, i.e., the diameter of proximal coronary arteries was less than 2 mm (see Figures 4B1, B2). Severe calcification of 8 coronary arteries may affect the accuracy of FFRCT values (see Figures 4C1, C2). Besides, a total of 26 coronary arteries were ICA negative but FFRCT positive.

## 4. Discussion

In this study, it was found that 85.4% of the ICA negative patients were also negative for FFRCT, and severe stenosis could be ruled out anatomically and functionally. In other words, most patients with ICA negative did not require further invasive FFR. FFRCT showed a moderate consistence with ICA and demonstrated excellent diagnosis performance according to ICA. And FFRCT had statistically significant effect on ICA positivity. Besides, 14.6% patients with mild to moderate coronary stenosis in ICA have functional ischemia according to FFRCT positive indications, indicating that although coronary stenosis did not meet the traditional revascularization standards, these patients had functional ischemia and should be revascularized to reduce the future incidence of cardiovascular events. However, for severely calcified, occluded, or small coronary arteries, the calculation of the FFRCT value was not reliable, so treatment strategy should be selected based on ICA in combination with clinical practice and QCA can be employed to provide an objective and independent parameter for the assessment of stenosis severity. Two-dimensional (2D) QCA was adopted in this study. 2D QCA assumed that the coronary cross-section was circular, while the coronary cross-section after three-dimensional (3D) reconstruction is oval. In theory, the vascular stenosis rate after 3D reconstruction is generally lower than that of 2D. However, Xu et al. (16) found that there was no statistical difference between 3D QCA and 2D QCA in measuring area stenosis rate. In addition, the ICA in this study was performed by cardiologists with more than 8 years of experience through multi-position and multi-angle projection, and the narrowest angle was found to calculate the coronary artery area stenosis rate, so our ICA results are also quite reliable. The lack of 3D QCA may be a deficiency of this study, which will be remedied by further work.

Curzen et al. (17) showed that, after FFRCT data became available, a change in the allocated management category on the basis of CCTA alone was seen in 36% cases. The randomized CRESCENT trials (18) found positive FFRCT in 51% patients. The availability of FFRCT would have reduced the number of patients requiring additional testing by 57% compared with CCTA alone. Reserving ICA for patients with a FFRCT ≤ 0.80 would have reduced the number of ICA following CCTA by 13%. Rabbat et al. (19) showed that compared to CCTA alone, CCTA and selective FFRCT reduced the rates of ICA (decline from 80 to 45%) for those with obstructive CAD. The PLATFORM study showed that, in patients with planned ICA, a diagnostic strategy based on FFRCT yielded a significantly lower rate of ICA showing no obstructive CAD (12.4%) than usual care (73.3%) (20). The ADVANCE study (21) showed that, FFRCT changed management recommendations from CCTA-based plans in approximately 70% subjects. An initial management decision for medical treatment was assigned to 790 cases, and this assignment remained unchanged after FFRCT in 93% of cases, with only 5.4% changing to revascularization. In the present study, the discordance rate of the risk stratification of FFRCT for ICA-positive lesions was 33.1% (91). With positive FFRCT as the reference standard, ICA was not required in these cases, which is consistent with the above study results.

In the study of Rabbat et al. (19), FFRCT of 31% patients with 25–49% stenosis was ≤ 0.8, and that of 55% patients with 50–69% stenosis was ≤ 0.8, suggesting that the degree of coronary stenosis may be negatively correlated with FFRCT value. Lossnitzer et al. (22) showed that in the group of patients with > 70% diameter stenosis, the FFRCT value on a per-lesion analysis was generally lower (mean: 0.72) than that with a 50–69% diameter stenosis (mean: 0.80) lesion. This is partially consistent with the results of our study. In the present study, FFRCT values decreased significantly with the aggravation of coronary stenosis, and for LAD and RCA, FFRCT values in patients with coronary stenosis ≥ 75% were significantly lower than those in patients with coronary stenosis < 75%, demonstrating that the more serious coronary stenosis showed, the lower the FFRCT value, and the more revascularization were required.

The conclusion of the present study is generally consistent with the above studies, but there are some differences, which may be due to the large number of the study population. And CT scanners are not completely consistent with previous studies. Besides, in this study, FFRCT is calculated using a basic deep learning commercial software, which is more accurate because of the incorporation of context information on target FFR along the vessel path and the workstation includes the neural networks set on each point of the vascular path. Structural and functional features of each point on the vascular centerlines are considered as input, while calculating FFR of each point as output. Therefore, it takes 5–10 min to calculate the FFRCT value of each patient, so the calculation is faster and more stable.

Besides, a total of 26 coronary arteries were ICA negative but FFRCT positive. In current clinical practice, most patients with negative ICA did not undergo further revascularization, which may delay the disease. Remarkably, FFRCT values of 22 (81.5%) coronary arteries in these patients were 0.75–0.80, that is, they were in the “gray zone,” and revascularization should be determined based on FFRCT, the severity of symptoms and the importance of coronary blood supply. Follow-up should be conducted to observe whether these patients, especially those in gray zones, had major cardiovascular events.

It is worth noting that DL-FFRCT has certain technical limitations. Firstly, when constructing the DL-FFRCT model, occluded or subtotal occluded coronary arteries often cannot be displayed, so FFRCT values at occluded distal ends cannot be calculated (as shown in Figures 4A1, A2). Secondly, if the coronary artery is too small, such as diameter < 2 mm, it is difficult to accurately find the coronary site with significant stenosis when constructing the DL-FFRCT model. This may be the reason why FFRCT values of 12 small LCX and 6 small RCA in this study are inconsistent with ICA results. In addition, coronary calcification has significant influence on diagnostic image quality and diagnostic accuracy (23, 24). In the present study, severe calcification of 8 coronary arteries were observed in cases whose ICA results were not consistent with FFRCT, we suppose that severe calcification may affect the accuracy of FFRCT values. Future studies need to calculate quantitative data such as coronary artery diameter and coronary artery calcification score.

Our study has some limitations: First, we do not use invasive FFR as gold standard. Second, this study was a single-center retrospective study with a large proportion of male patients. Third, at the time of enrollment in this retrospective study, 85/314 (27%) of patients were excluded due to motion artifacts that could not calculate the FFRCT value.

Fourth, no follow-up was conducted to determine whether the treatment strategy based on DL-FFRCT would affect the occurrence of major adverse cardiovascular events. The clinical significance of DL-FFRCT should be further evaluated by multi-center prospective studies and further the sample size will be enlarged. In addition, coronary calcification scores should be calculated for all patients in this study to more accurately assess the effect of coronary calcification on FFRCT.

This study revealed that most patients with ICA negative did not require further invasive FFR. It’s worth noting that some patients with mild to moderate coronary stenosis of CCTA and ICA may also have functional ischemia. Combined with DL-FFRCT, CCTA can better guide subsequent treatment, thus reducing the incidence of major adverse cardiovascular events. However, for severely calcified, occluded, or small coronary arteries, treatment strategy should be selected based on ICA in combination with clinical practice.

## Data availability statement

The original contributions presented in this study are included in the article/supplementary material, further inquiries can be directed to the corresponding authors.

## Ethics statement

The studies involving human participants were reviewed and approved by the Ethics Committee of Tongji Hospital Affiliated to Tongji Medical College, Huazhong University of Science and Technology. The patients/participants provided their written informed consent to participate in this study.

## Author contributions

YP: concept, design, data acquisition, data analysis, statistical analysis, and manuscript editing. TZ: definition of intellectual content, literature search, clinical studies, and statistical analysis. YW: data acquisition, data analysis, and manuscript preparation. YD: data analysis, statistical analysis, manuscript preparation, and manuscript editing. HG: concept, design, literature search, and manuscript review. All authors contributed to the article and approved the submitted version.
